# 

**Published:** 1950-07

**Authors:** 


### Local Medical Notes

---

### Honours

Professor F. Blakemore has received from the University of Liverpool the degree of D.V.Sc. *Honoris Causa.*

**The XXXIX Long Fox Memorial Lecture** will be given in the University by Sir William G. Savage, M.D., sometime Medical Officer of Health for Somersetshire; title " **Some Food Infection Problems** ".

The next **Carey Coombs Memorial Lecture** will be delivered at 8.30 p.m. on November 14th, 1950, by Dr. Maurice Campbell, *O.B.E.*, D.M., F.R.C.P., Subject: " **Aspects of Congenital Heart Disease** ".*

### Appointments

Dr. L. B. Thomas.—Associate Professor of Neuro-Physiology and Director of Department of Electro-encephalography, University of Washington, U.S.A.

Dr. A. J. Keevill, M.B.E., M.B.—Regional Assistant Director of Medical Services, Tanganyika.

Mr. J. F. Bourdillon, F.R.C.S.—Consultant Orthopaedic Surgeon, North Gloucestershire clinical area.

Mr. T. Jackson, F.R.F.P.S.—Dental Surgeon, North Gloucestershire clinical area.

Mr. E. H. Rainer, F.R.C.S.—Consultant Surgeon for diseases of Ear, Nose and Throat, Dartford and Sidcup area, S.E. Metropolitan Region.

Dr. W. H. K. Carpenter.—Deputy Medical Superintendent, Stoke Park Colony, Bristol.

Dr. B. P. Griffin,—Deputy Medical Superintendent, Hortham Colony, Bristol.

Dr. R. E. Steiner, M.B., D.M.R.—Radio diagnostician, Hammersmith Hospital, London.

*United Bristol Hospitals—*

Dr. V. T. Baxter, Dr. J. H. Challenger.—Consultant Anaesthetists.

Mr. H. K. Lucas, *M.B.E.*, M.Ch., F.R.C.S.; Mr. M. P. McCormack, M.B., Ch.B., F.R.C.S.—Consultant Orthopaedic Surgeons.

* By an unfortunate error these two announcements became telescoped into one in our last issue.

Dr. H. M. Golding, Mr. J. R. Nicholson-Lailey and Dr. S. N. Scott have been elected Members of Council of the B.M.A.

Professor R. Milnes Walker has been appointed Vice-President of the Section of Surgery for the Annual Meeting of the B.M.A. in July.

### University of Bristol: New Veterinary Buildings

Veterinary education is a new development in the University: the first degree students were admitted in 1949. Many of the classes will be taken in existing departments. But to meet the needs of the veterinary student it was necessary to provide a new building in Bristol for pre-clinical studies, and accommodation at Langford for the purely vocational side of the course.

The new Veterinary School building in Bristol is located in Park Row. It was completed at the beginning of May and is a substantial addition to the buildings on the University site. Special consideration has been given to the needs of anatomy and physiology. On the ground floor there is a large dissecting laboratory, leading from a receiving room, with an accessible cold room large enough to hold intact carcases. There is also an anatomy preparation room and an X-ray unit. All the rooms in which large animals are likely to be dealt with are linked by an overhead runway, to facilitate the movement of carcases. The lecture theatre is conveniently placed and the building contains research laboratories and a museum. The first floor contains a photographic room and laboratories for physiology, biochemistry, histology, and embryology, with adjoining preparation rooms. Although it is not intended that animals should be housed for long periods, there is accommodation at the back of the building for animals both large and small.

### Scholarships and Prizes

### SCHOLARSHIPS

*Lady Haberfield.*—D. W. Trump.

*Sanders.*—W. M. Dutton.

### PRIZES

*Marshall and Clarke.*—W. G. Benson.

*Russell Cooper.*—W. G. Benson.

*Carey Coombs*—Dr. I. McD. Stewart.

*Edward Fawcett.*—A. J. Webb.

*Henry Clarke.*—W. M. Dutton.

*Medical Suple.*—M. D. Turner.

*Surgical Suple.*—E. M. W. Houghton.

*Barrett Rouè.*—S. J. Lloyd.

*Mackie.*—M. B. Turner.

*Phillips.*—E. Joseph.

*Richard Clarke.*—Z. Fluss.

*Western Dental.*—T. F. Harris.

*Attenborough Medal.*—T. F. Harris.

*Foyle.*—A. J. Webb.

*Augustin Prichard.*—Miss D. Ellis, Miss G. M. Tebbutt (*aequales*).

### Degrees and Diplomas

**University of Bristol.**—

M.B., Ch.B. (*with Second Class Honours*) R. J. Ancill, W. A. W. Dutton,* M. D. Turner.†

M.B., Ch.B.—P. J. D. Benjafield, H. J. Bland, Margaret H. Buston,‡ E. R. Duchesne, F. V. Eastman, D. A. Ellis, C. C. Ewing, Z. Fluss, J. C. Gregg, R. Hill, E. A. W. Houghton, G. A. K. M'C. Jahans, Megan D. M. Johnson, H. I. Leigh,† G. Lucas, Jean M. Menzies, G. W. B. Peel, Barbara M. Philpott,† L. P. Spence, L. A. D. Tovey, Joan Walker.

B.D.S.—D. C. Berry, J. S. Cooper.

L.D.S.—A. H. Chivers, E. K. Joseph, J. J. Tittle.

D.P.H.—A. J. Board, E. L. Fawssett.

D.P.M.—M. W. Annear, J. A. Bickford, R. M. Ellison.

M.B., Ch.B.—*Second Examination:* Prudence M. Beckingham, Isabel K. E. Brister, Margaret E. Chesterfield, K. G. Collins, M. L. Cox, J. H. L. Griffin, M. J. Page, M. J. Ree, E. C. Skeens, Catherine M. Stephen.

**Royal College of Physicians.**—F.R.C.P.—Dr. A. M. G. Campbell.

**Royal Colleges of Physicians and Surgeons (Conjoint Board).**—M.R.C.S., L.R.C.P.: E. R. Duchesne.

**Royal College of Physicians, Edinburgh.**—M.R.C.P.: D. J. A. C. Wilson.

* Distinction in Obstetrics. † Distinction in Public Health. ‡ Distinction in Child Health.

